# Comparative Analysis of Direct Hospital Care Costs between Aseptic and Two-Stage Septic Knee Revision

**DOI:** 10.1371/journal.pone.0169558

**Published:** 2017-01-20

**Authors:** Richard Kasch, Sebastian Merk, Grit Assmann, Andreas Lahm, Matthias Napp, Harry Merk, Steffen Flessa

**Affiliations:** 1 Center for Orthopedics, Trauma Surgery and Rehabilitation Medicine, Clinic and Outpatient Clinic for Orthopedics and Orthopedic Surgery, University Medicine Greifswald, Greifswald, Germany; 2 Kliniken Maria Hilf Mönchengladbach, Academic Teaching Hospital of the RWTH Aachen, Mönchengladbach, Germany; 3 Center for Orthopedics, Trauma Surgery and Rehabilitation Medicine, Department of Trauma Surgery, University Medicine Greifswald, Greifswald, Germany; 4 Department of Health Care Management, Faculty of Law and Economics, Ernst-Moritz-Arndt-University, Greifswald, Germany; Royal Prince Alfred Hospital, AUSTRALIA

## Abstract

**Background:**

The most common intermediate and long-term complications of total knee arthroplasty (TKA) include aseptic and septic failure of prosthetic joints. These complications cause suffering, and their management is expensive. In the future the number of revision TKA will increase, which involves a greater financial burden. Little concrete data about direct costs for aseptic and two-stage septic knee revisions with an in depth-analysis of septic explantation and implantation is available.

**Questions/Purposes:**

A retrospective consecutive analysis of the major partial costs involved in revision TKA for aseptic and septic failure was undertaken to compare 1) demographic and clinical characteristics, and 2) variable direct costs (from a hospital department’s perspective) between patients who underwent single-stage aseptic and two-stage septic revision of TKA in a hospital providing maximum care. We separately analyze the explantation and implantation procedures in septic revision cases and identify the major cost drivers of knee revision operations.

**Methods:**

A total of 106 consecutive patients (71 aseptic and 35 septic) was included. All direct costs of diagnosis, surgery, and treatment from the hospital department’s perspective were calculated as real purchase prices. Personnel involvement was calculated in units of minutes.

**Results:**

Aseptic versus septic revisions differed significantly in terms of length of hospital stay (15.2 vs. 39.9 days), number of reported secondary diagnoses (6.3 vs. 9.8) and incision-suture time (108.3 min vs. 193.2 min). The management of septic revision TKA was significantly more expensive than that of aseptic failure ($12,223.79 vs. $6,749.43) (p <.001).

On the level of the separate hospitalizations the mean direct costs of explantation stage ($4,540.46) were lower than aseptic revision TKA ($6,749.43) which were again lower than those of the septic implantation stage ($7,683.33). All mean costs of stays were not comparable as they differ significantly (p <.001). Major cost drivers were the cost of the implant and general staff. The septic implantation part was on average $3,142.87 more expensive than septic explantations (p <.001).

**Conclusions:**

Our study for the first time provides a detailed analysis of the major direct case costs of aseptic and septic revision TKA from the hospital-department’s perspective which is the basis for long-term orientated decision making. In the future, our cost analysis has to be interpreted in relation to reimbursement estimates. This is important to check whether revision TKA lead to a financial loss for the operating department.

## Introduction

### Background

While the number of primary total knee arthroplasties (TKA) has already reached a high level in some western industrialized countries, global data suggest that other countries are quickly catching up [1–6]. Due to this trend and an ageing society it is expected that especially the rate of revision TKA surgery will increase disproportionately in the next decade [7–9]. Revision TKA and its possible complications require more resources than primary TKA in terms of diagnostic procedures, length of hospital stay and postoperative care [10–13]. This applies even more to septic revision procedures than aseptic prosthesis failure [14–16]. The high number of resource-intensive interventions that are expected to be required in the future, along with increasing economic pressure on hospitals and health systems in general, place revision TKA surgery in the focus of economic discussions.

Aseptic failure is one of the main reasons why patients may require long-term revision after TKA and is managed by arthroplasty of the loose joint parts [17,18]. A more devastating complication is chronic infection of knee arthroplasties, which is associated with higher mortality, morbidity and health care use [9]. From a clinical perspective, two-stage revision is considered the benchmark procedure, as it requires extraordinary medical expertise and care and should be performed by experienced maximum care providers [17,19–23]. Even from the hospital department’s perspective, septic revision TKAs are not only clinically challenging but also economically outstanding as they are non-elective, time-consuming surgeries, requiring expensive prostheses and medications, longer hospital stays including ICU, and involve more complications [15].

Patient management has more and more been affected by financial restrictions [15,24–27]. Most hospitals provide primary care, performing a wide range of different basic procedures. In addition, maximum care providers such as university hospitals are responsible to offer specialized treatment for rare diseases and life-threatening conditions. Optimally, the total revenues of a hospital from a mixture of basic and specialized medical services should exceed its costs [28].

For a hospital to make sound economic decisions, it is necessary to analyze variable and case-fixed costs. Reimbursement is not a sound basis for decision making, as the reimbursement systems differ between countries and cannot be of influence by the hospital. For orthopedic departments it is essential to know where and what costs occur in the treatment of an individual patient to have a chance to restructure workflow or use synergies.

Healthcare providers tend to regard different types of joint replacement procedures together without adequately considering differences between primary and revision THAs and TKAs although these differences have implications for patient management and resource allocation. To the best of our knowledge, all published cost studies of revision TKA, unlike those of aseptic and septic total hip revision, use a total cost approach [29–31], which provides no basis for economic decisions for the department performing this kind of surgery [15,26,32–36]. In contrast, the partial cost approach we present here and have already used for a similar study of hip replacement distinguishes variable from case-fixed costs [30]. It thus contributes essential data for making future management decisions in hospital departments. Furthermore, to the best of our knowledge, a comprehensive cost assessment of two-stage septic knee revisions with an in depth-analysis of septic explantation and implantation has not been done before. In addition, our review of the literature has not revealed any publication comparing costs of aseptic and septic revision TKA using this approach.

### Questions/Purposes

The purpose of our analysis was to evaluate the hypothesis that explantation and implantation procedures of a septic two-stage knee revision are more expensive than the aseptic (one-stage) procedure using an approach that analyzes those costs that are important for hospital decision-makers. To avoid the shortcomings of earlier publications addressing this question, we compare variable and case-fixed costs in a series of consecutive patients who underwent single-stage aseptic and two-stage septic revision of TKA from a hospital department’s perspective. We separately identify the major cost drivers of knee revisions.

## Materials and Methods

### Study design and patient selection

The local ethics committee of Ernst-Moritz-Arndt University Greifswald (Felix-Hausdorff-Str. 3, 17487 Greifswald) approved the study (BB 010/13) and waived written informed consent, without restrictions on data. In the methods section we followed previously published protocols, describing in detail design, patient selection, outcome measures and the calculation of costs [24,30]. All patients (n = 106) undergoing revision TKA were treated in a university hospital providing primary medical care and specialized procedures in the period of 39 months ending Jan. 1, 2012. Since our aim was to analyze all aseptic as well as septic revision TKA´s performed, no case exclusion was done in this consecutive study. Following international guidelines, and cost studies of the hip published before [30], patients were included when (1) they had an ICD-10 (International Classification of Diseases) indication for TKA revision according to Code T84.0 (aseptic—mechanical complication of internal joint prosthesis) or T84.5 (septic—infection and inflammatory reaction due to internal joint prosthesis) and (2) underwent procedures with OPS (Operation and Procedure) Codes 5–823 (Re-operation, exchange or removal of an artificial knee joint) and 5–822 (Implantation of an artificial knee joint) [37,38]. The inclusion of patients with aseptic/septic failure was based on the presence of: (1) typical clinical signs and symptoms in the leg; (2) imaging confirmation of loosening including x-rays and scintigraphy; and (3) characteristic laboratory and pathophysiologic values in blood [39,40]. All patients with septic TKA were tested for infection via joint biopsy. A positive microbiological culture was the indication for the periprosthetic infection, knowing that there are also septic joints which are culture negative. Since our main question of interest focused on the average TKA replacement we consecutively included all aseptic cases independently of the revision extent. Nevertheless we tested whether mean costs of total revision cases differ from those of partial aseptic revisions to make sure that we provide a valid comparison.

As described in detail previously, “All data were systematically generated using the unique case number to search the hospital’s health information system such as SAP. When the electronic patient file was incomplete or inconsistent with regard to eligibility, or patient outcome, we additionally retrieved the paper files from the hospital’s archive to obtain more data [24,30]”.

The local ethics committee of Ernst-Moritz-Arndt University Greifswald approved the study (BB 010/13) and waived written informed consent.

### Demographic and clinical outcome measures

Following Assmann et al. and Kasch et al. patient demographics (age, gender), clinical measures (pathogens, length of stay, operative time, number of comorbidities, extent of revision), consumption of hospital resources, and economic data were retrospectively evaluated case-by-case [24,30]. Following international guidelines septic TKA revisions are performed as two-stage procedures with two hospital stays, which we analyzed separately (septic explantation versus septic implantation) [17,18]. While adding both TKA operations together when comparing septic versus aseptic knee revision, we also utilized the separate data, amongst others, for the comparison of septic implantation (e.g., stage two alone) with aseptic revision.

### Determination of costs

The study took place in Germany, and therefore all economic data were obtained in Euro (€). Subsequently, all quotations were converted to United States Dollars (US$), with US-$1 equivalent to €0.9006, with regard to the mean international exchange rate in 2015.

As described in detail previously the analysis included variable and case fixed costs, following Kasch et al. [24,29,31,41]. “Those costs could be directly related to an individual case and can be influenced directly. We did not include fixed hospital costs that arise irrespective of the treatment of individual patients (energy, heating, license fees for software, room costs, staff costs for security, gardening, cleaning). Fixed costs are excluded because they are not influenced by the treatment of patients and hence are not relevant for managerial decision-making” [30].

Costs were analyzed using the outcome measures presented in Table 1. These were included in terms of frequency while patient’s stay in the hospital to then assign the costs they generated. To ensure we analyze realistic cost structures we utilized the hospital’s actual purchase prices rather than official list prices. Especially noteworthy is the calculation of drug costs. According to the recorded admission medication we analyzed every single day of hospital stay by evaluating all information of the patient file. In cooperation with the university hospital pharmacy we searched appropriate price data for more than 400 different drugs.

**Table 1:** Performance and cost areas for the analyzed cases. Following Kasch et al. [24].

Location of cost generation	Cause of costs	Unit	Cost per unit [$]	Costs [$]
**Normal ward**				
Nursing staff	Nursing staff time	[ppr min]	Costs per min	Time required * costs per unit of time
Laboratory	Laboratory diagnostics	[Points]	Ø Point value	Points * Point value
Radiology	Radiological diagnostics	[Points]	Ø Point value	Points * Point value
Physiotherapy	Physiotherapy	[Points]	Ø Point value	Points * Point value
Medication	Medication during whole stay	[**$**]	[**$**]	Direct costs
Intensive care	ICU treatment	[min]	Costs per min	Time required * costs per unit of time
**Surgical costs**				
Orthopedics (doctor)	Orthopedic surgeon time	[min]	Costs per min	Time required * costs per unit of time
Orthopedics (nursing)	Surgical nursing staff time	[min]	Costs per min	Time required * costs per unit of time
Anesthesiology (doctor)	Anesthesiologist time	[min]	Costs per min	Time required * costs per unit of time
Anesthesiology (nursing)	Anesthesiological nursing staff time	[min]	Costs per min	Time required * costs per unit of time
Implant	Implant	[**$**]	[**$**]	Direct costs
Expendable material	Materials used	[**$**]	[**$**]	Direct costs
Sterilization	[sterilization sieve]	Costs per sterilization sieve	Number of sterilization sieves *costs per sterilization of one sieve	
Blood products	Blood transfusions	[**$**]	[**$**]	Direct costs

* = multiplication sign

As we expected ICU to be an important cost factor we included this cost data as well. Therefore, we got minute exactly duration information about the patient’s stay on the ICU. According to the central controlling business unit of the analyzed hospital the average costs of 24 hours on the ICU were $1,296.86.

### Statistical methods

Statistical methods are in line with our former published analyses of the hip: “All data are given as means with standard deviations. As cost data are often not normally distributed, but skewed we tested this (Kolmogorow-Smirnow-test) and used for the skewed data non-parametric tests as the Mann-Whitney U-test. For normally distributed data Chi^2^-test and t-test, assuming statistical significance at p <.05. Statistical analysis of all parameters was undertaken using SPSS v.19” [30].

## Results

### Demographic and clinical results

One hundred and six patients undergoing revision of TKA due to septic (35 cases) or aseptic (71 cases) reasons were included in this analysis. Patient demographics are shown in Table 2. While aseptic cases require one hospital stay (median: 15.0 days), all septic cases are associated with at least two separate operations (mean: 2.0, min: 2, max: 5) on two separate hospital stays (explantation of the infected endoprosthesis (median: 18.0 days) and implantation of the new joint (median: 15.0 days)).

**Table 2:** Baseline demographic and clinical characteristics of patients who underwent TKA categorized by indication. Continuous data are mean (SD), categorical data are counts (%).

	Aseptic	Septic^1^	p-value	All cases
Total	71 (67.0%)	35 (33.0%)	-	106 (100%)
Complete^2^	64 (90.1%)	35 (100.0%)	-	99 (93.4%)
Partial^3^	7 (9.9%)	0 (0.0%)	-	7 (6.6%)
Age [years]	65.6 (9.4)	69.3 (9.3)	.034	66.8 (9.5)
Gender			.137	
Male	22 (31.0%)	16 (45.7%)	-	38 (35.8%)
Female	49 (69.0%)	19 (54.3%)	-	68 (64.2%)
Number of diagnoses	6.3 (3.4)	9.8 (3.6)	<.001	7.4 (3.9)
Number of operations^4^	1.0 (0)	2.0 (1.0)	<.001	1.5 (0.7)
Length of stay [days]^4^	15.0 (1.0)	34.0 (11.0)	<.001	23.3 (16.1)
Explantation	-	18.0 (9.0)	-	-
Implantation	-	15.0 (6.0)	-	-
Pathogenic agent^5^				
*Staphylococci*	-	35	-	35
*Streptococci*	-	4	-	4
*Others*^6^	-	3	-	3

^1^ Two hospital stays;

^2^ Revision of all components;

^3^ The 7 aseptic partial revision surgery cases were done as femur only: 4 (5.6%) and tibia only: 3 (4.3%);

^4^ As “Number of operations” and “Length of stay” are not normally distributed medians and interquartile ranges are presented

^5^ 30 patients with one pathogenic agent, 4 patients with 2 pathogenic agents, 1 patient with 4 pathogenic agents,

^6^ Escheria coli, Enterobacter cloacae, Clostridium perfringens

We found sex distribution to be the same with no statistical difference (see Table 2). To the disadvantage of septic cases the mean patient age at the time of revision surgery, number of documented comorbidities, the mean hospital stay and the mean number of operations differed significantly to aseptic patients. Histological examinations were performed in all septic cases. The range of pathogenic agents isolated in patients undergoing septic revision is even shown in Table 2.

Total revision of all components was done in 64 of the 71 (90.1%) aseptic cases, while this was done in all septic cases (n = 35, 100%). The mean overall costs did not differ in both aseptic groups (p = .600). Therefore, we compared all aseptic cases (n = 71) with the septic treatments of total revision using two revision approach subsequently.

### Health care resource utilization and cost

Table 3 lists the resources utilized and services provided during patient´s hospital stay(s) on normal ward. In all aseptic (septic) cases, a mean of 25.9 (41.5) physiotherapy units per patient were exercised and 1.9 (4.4) radiological imaging tests were performed. Furthermore, determination of 10 (35) laboratory parameters were required.

**Table 3:** Average diagnostic and treatment resource consumption separately for aseptic and septic hospital stay and categorized by explantation and implantation procedure. Data are mean (SD).

	Aseptic (n = 71)	Septic (n = 35)^1^	p-value	Septic explantation (n = 35)	Septic implantation (n = 35)	p-value	All cases (n = 106)
Radiology	1.9 (1.2)	4.4 (3.7)	<.001	2.6 (3.5)	1.7 (1.1)	<.645	2.8 (2.6)
Laboratory	10.0 (15.6)	35.3 (34.8)	<.001	22.4 (24.1)	12.9 (13.6)	<.033	18.4 (26.4)
Drugs	7.1 (3.3)	16.1 (4.6)	<.001	8.1 (3.1)	8.00 (2.2)	<.864	15.5 (4.6)
Physiotherapy	25.9 (7.3)	41.5 (18.1)	<.001	18.4 (12.5)	23.1 (12.2)	<.025	31.1 (14.0)

^1^ Two hospital stays

As shown in Table 4, over half of the costs in the normal ward are accounted for nursing care followed by physiotherapy. Mean nursing costs in the ward were greater for septic explantations at $1,525.75 (SD: 640.62) than for septic implantations at $1,021.02 (SD: 438.28), which was statistically significant. Mean medication costs per patient were significantly higher in the septic cases (p <.001), at $98.42 (SD: 56.50) for daily medication and $123.53 (SD: 87.04) for antibiotics mostly (76.1%) consumed during time of septic explantation ($94.07, SD: 76.09).

**Table 4:** Average diagnostic and treatment costs separately for aseptic and septic hospital stay and categorized by explantation and implantation procedure. Data are mean [$] (SD).

	Aseptic (n = 71)	Septic (n = 35)^1^	p-value	Septic explantation (n = 35)	Septic implantation (n = 35)	p-value	All cases (n = 106)
Nursing Normal Ward	777.68 (243.10)	2,546.77 (885.66)	<.001	1,525.75 (640.62)	1,021.02 (438.28)	<.001	1,361.81 (986.54)
Radiology	42.46 (42.01)	148.03 (261.02)	<.001	108.56 (254.00)	39.47 (38.88)	<.518	77.31 (160.40)
Laboratory	36.77 (40.05)	212.03 (221.83)	<.001	154.53 (208.63)	57.50 (47.74)	<.001	94.64 (154.47)
Drugs	43.31 (33.76)	221.95 (112.41)	<.001	140.87 (93.09)	81.08 (53.90)	<.001	102.29 (109.44)
Physiotherapy	354.45 (95.67)	578.52 (236.08)	<.001	262.10 (162.88)	316.42 (166.72)	<.055	428.43 (188.04)

^1^ Two hospital stays

The mean incision-suture time was 108.3 min in aseptic revisions versus 193.2 min in two-stage septic revisions. In aseptic (septic) procedures, a mean of 12 (18) sterilization sieves and 1 (5) blood units were required (see Table 5). In the group of septic knee revision procedures, 26 patients (74.3%) were in the ICU for at least a couple of hours. In contrast, only 12 of the 71 patients with aseptic revision (16.9%) required ICU treatment.

**Table 5:** Average surgical consumption (including Intensive Care Unit) separately for aseptic and septic hospital stay and categorized by explantation and implantation procedure. Data are mean (SD).

	Aseptic (n = 71)	Septic (n = 35)^1^	p-value	Septic explantation (n = 35)	Septic implantation (n = 35)	p-value	All cases (n = 106)
Incision-suture time [min]	108.3 (28.8)	193.2 (48.0)	<.001	81.9 (36.1)	111.3 (28.9)	<.001	136.3 (53.9)
Sterilization[No. of sieves]	12.1 (4.3)	17.8 (4.7)	<.001	7.4 (3.7)	10.4 (4.9)	<.006	13.9 (5.2)
Blood units	1.4 (1.6)	5.2 (6.6)	<.001	2.8 (5.0)	2.4 (2.3)	<.596	2.7 (4.3)
ICU^2^ duration [days]	0.2 (0.4)	1.5 (1.8)	<.001	0.9 (1.4)	0.5 (0.7)	<.328	0.6 (1.2)

^1^ Two hospital stays,

^2^ Intensive Care Unit

The mean staff cost for surgery was $828.15 (SD: 171.44) for aseptic procedures, which was $944.70 significantly lower than the mean staff cost of septic intervention ($1,772.85, SD: 552.25) (p <.001). The mean implant costs in septic procedures was $4,208.28 (SD: 1,797.23), which was higher, but not statistically significant, than the mean implant cost of $3,941.21 (SD: 1,069.84) in aseptic procedures (p <.137). All other surgery related costs differed significantly between aseptic and septic cases (see Table 6).

**Table 6:** Average surgical costs (including Intensive Care Unit) separately for aseptic and septic hospital stay and categorized by explantation and implantation procedure. Data are mean [$] (SD).

	Aseptic (n = 71)	Septic (n = 35)^1^	p-value	Septic explantation (n = 35)	Septic implantation (n = 35)	p-value	All cases (n = 106)
**Staff Costs**							
Orthopedics (doctor)	394.70 (81.15)	821.59 (209.79)	<.001	387.68 (186.89)	433.91 (89.62)	<.001	535.66 (243.58)
Orthopedics (nursing)	160.95 (32.24)	334.72 (90.22)	<.001	161.00 (78.48)	173.72 (42.68)	<.007	218.33 (100.35)
Anesthesiology (doctor)	160.98 (35.79)	365.71 (160.45)	<.001	185.40 (145.31)	180.31 (49.18)	<.030	228.58 (136.19)
Anesthesiology (nursing)	111.52 (22.25)	250.81 (91.79)	<.001	128.02 (84.95)	122.80 (27.88)	<.060	157.51 (85.97)
**Material Costs**							
Implant	3,944.21 (1,069.84)	4,208.28 (1,797.23)	<.137	16.19 (82.05)	4,192.09 (1,807.35)	<.001	4,031.40 (1,351.00)
Expendable Material	57.57 (32.52)	136.95 (60.25)	<.001	73.74 (39.17)	63.22 (38.77)	<.254	83.78 (57.34)
Sterilization	270.83 (106.10)	406.70 (117.35)	<.001	161.06 (88.23)	245.65 (123.83)	<.002	315.69 (126.84)
Blood units	145.11 (163.91)	543.79 (663.29)	<.001	280.19 (492.22)	263.61 (275.52)	<.571	276.76 (442.56)
**ICU**^2^	178.77 (656.98)	1,039.86 (1,515.51)	<.001	686.14 (1,200.67)	353.73 (516.90)	<.277	463.09 (1,094.08)

^1^ Two hospital stays,

^2^ Intensive Care Unit

**Fig 1:**
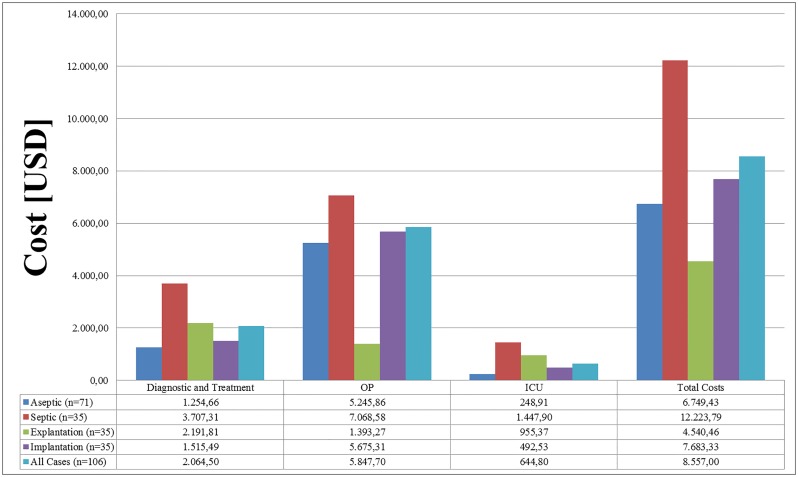
Bar diagram of total costs for aseptic procedures (n = 71), septic procedures (n = 35), septic explantations (n = 35), septic implantations (n = 35), and total procedures (n = 106) [$]. Septic (n = 35) = septic explantations (n = 35) + septic implantations (n = 35). Diagnostic and Treatment: Nursing Normal Ward + Radiology + Laboratory + Drugs + Physiotherapy. OP: Personal Costs + Implant + Expendable Material + Sterilization + Blood units. Total Costs: Diagnostic and Treatment + OP + ICU. ICU = Intensive Care Unit.

Fig 1 provides a bar diagram of costs. The mean direct cost for patients undergoing revision TKA was $10,234.42 (SD: 4,519.65). The average cost difference between aseptic procedures (mean: $8,072.51, SD: 1,847.15) and septic procedures (mean: $14,620.00, SD: 5,147.85) was $6,547.49 and statistically significant (p <.001).

The mean cost of septic explantations was $5,430.52 (SD: 3,948.81) versus $9,189.48 (SD: 2,714.97) for septic implantations, which is in addition 81.1% more than the cost of aseptic procedures. The mean cost of complete (revision of all components) aseptic revision TKA´s (n = 64) were $8,193.10 (SD: 1,728.21) and quite less expensive than septic implantation (difference: $996.38) (p = .014).

OP accounts for the largest component of the complete cost of knee revision ($5,847.70, SD: 1,473.68), followed by costs for diagnostic and treatment ($2,064.50, SD: 1,420.22) and ICU costs ($644.80, SD: 1,523.40). The largest share is accounted by the cost of the implant (47.1%), followed by normal ward staff cost (15.9%). Other major cost items are surgery staff and physiotherapy costs, accounting for 13.3% and 7.5%.

## Discussion

### Background and rationale

Over a very short period of time, TKA has evolved into a widely accepted treatment mostly for patients with arthrosis of the knee [10,34,42]. Factors that have contributed to this development include an aging population with more diseases involving the musculoskeletal system such as the knee as well as the improved clinical outcome of knee replacement after advancement of both the surgical procedure and implants over the last decades [7,9,11,35]. As already shown for other joint replacement operations, such as the hip, revision operations after knee arthroplasty are more demanding than primary TKA, resulting in longer mean operation time, more blood transfusions, longer ICU stays due to higher complication rates, and a longer mean hospital stay [5,43]. The implants used in revision operations are also more complex and expensive. Therefore, it is obvious that the cost of (two-stage) revision TKA exceeds that of primary TKA [14,44]. Available studies have investigated hospital expenses and revenues for revision TKAs by using cost-effective analysis or a total cost approach [5,10,15,22,25–27,32,34–36,42,45].

Our study is the first to analyze the cost of aseptic and septic revision TKA using a partial cost approach. This approach allows us to identify the main cost drivers, which are relevant for future-orientated decision making. On the other hand, our approach ignores reimbursement, which cannot be influenced by an individual hospital, and also excludes fixed costs because they cannot be changed by a hospital department on a short-term basis.

### Study limitations

Our study has some limitations (see [30]): as our study was conducted at a single university hospital, our study population might not be representative for other hospitals or centers performing aseptic and septic knee revision surgery. Even prices for materials (e.g. implants) and salary structures differ a lot between countries and health care systems. Nevertheless, we think that relative differences in costs for aseptic and septic revision TKAs are similar across institutions. Our evaluation was limited by a small number of patients undergoing septic revision. Nevertheless, the total sample size was large enough for statistical analysis. We only analyzed direct costs arising in the hospital performing the operations. For a comprehensive assessment, future studies should additionally analyze the cost of outpatient aftercare and rehab related to revision TKA.

### Interpretation of results

The direct comparison of aseptic revision TKA (n = 71, $6,749.43) with septic revisions (n = 35, $12,223.79) confirms our hypothesis that the septic procedure is more costly than the aseptic one (p <.001). This is mainly justified by two separate hospital stays and operations during septic revisions. Nevertheless even factors like the number of comorbidities may have an influence on the overall costs. Comparisons with previously published data should be regarded with caution as analytical approaches and health care systems differ. Differences also exist with regard to operative techniques, the implants used, and additional measures (e.g., intravenous vs. oral antibiotic treatment), all of which may affect costs directly or indirectly (shortening of hospital stay). Oduwole et al. reported costs of $13,665.70 for aseptic and $20,815.57 for septic TKA revisions performed between 2002 and 2006 [5]. To make costs comparable we converted cost data presented here using the mean international exchange rate of 2015. In comparison to our results the total difference appears very high, especially as nursing and theater costs were not included, thus costs seem to be underestimated. We assume that our use of negotiated prices including rebates rather than list prices might be a reason for this. Nevertheless, our presented outcome is in line with previous results which reported significantly lower costs for revisions of aseptic total hip arthroplasty (THA) compared with septic revision of THA ($4,588.02 vs. $12,022.95 vs.) (p <.001) [30]. To make costs comparable we converted cost data presented here (on the basis of exchange rates in 2013) using the mean international exchange rate of 2015. While costs for septic revisions are nearly the same (-$200.84) there is a remarkable difference (+$2,161.41) for aseptic revision TKAs. This might be due to differences in the proportion of complete revisions: In contrast to the published data about revision THA´s (17%) our aseptic TKA cases included more than 90% of complete revisions. Another reason might be that implant costs of TKA were higher independently from complete or partial revision.

The higher cost of septic TKA revisions is mainly attributable to higher staff involvement and implant costs. All in all, septic TKA failure is not only a more complex life-threatening intervention but also incurs a markedly higher use of blood units, antibiotics and physical therapy with associated higher cost.

As expected, our separate analysis of the costs of both hospital stays of septic knee revision procedures confirms earlier studies reporting significantly lower mean cost of septic explantations ($4,540.46) versus septic implantations ($7,683.33) (p <.001) [30].

The cost difference between the aseptic cases and septic implantation is rather low ($933.90) but statistically significant (p = 0.008). This is quite interesting because our earlier cost analysis study found a much higher difference for revision THA (difference: $2,829.86) [30]. Even the comparison of the completely aseptic cases (n = 64, $6,850.25) and septic implantation (n = 35, $7,683.33), as a kind of sensitivity analysis, confirms our result (p = 0.014). Cost differences might be attributable to a longer hospital stay (15.2 vs 18.4 days), higher implant costs ($3,944.21 vs. $4,192.09) and a greater resource utilization, e.g. for blood units ($145.11 vs. 263.61) or drugs ($43.31 vs. $81.08). This clearly indicates that septic revisions are significantly more expensive, reflecting the fact that they are clinically more challenging and also a higher burden for patients.

## Conclusions

Our study for the first time provides a detailed analysis of the major direct case costs of aseptic and septic revision TKAs arising for the hospital department performing this kind of surgery. Our analysis provides hospitals with a sound basis for long-term economic planning. We found the costs to be nearly twice as high for septic versus aseptic revisions. In the two-step septic revision procedure, costs of septic explantation differ significantly from those of the implantation procedure. Implant costs and general staff costs were identified to account for the largest proportion of overall costs. Comparisons with data on THA replacement show that there are relevant differences in cost structure, thus decision-making should not be based on generalized assumptions based on one type of procedure.
